# Production of theophylline via aerobic fermentation of pu-erh tea using tea-derived fungi

**DOI:** 10.1186/s12866-019-1640-2

**Published:** 2019-11-26

**Authors:** Binxing Zhou, Cunqiang Ma, Xiaoying Ren, Tao Xia, Xiaohong Li, Yang Wu

**Affiliations:** 1 College of Long Run Pu-erh Tea, Yunnan Agricultural University, Kunming, 650201 Yunnan People’s Republic of China; 2 Henan Key Laboratory of Tea Comprehensive Utilization in South Henan, Xinyang Agriculture and Forestry University, Xinyang, 464000 Henan People’s Republic of China; 3 Liaocheng Senior Financial Vocational School, Liaocheng, 252000 Shandong People’s Republic of China; 4 State Key Laboratory of Tea Plant Biology and Utilization, Anhui Agricultural University, Hefei, 230036 Anhui People’s Republic of China

**Keywords:** Pu-erh tea, Aerobic fermentation, Fungi, Caffeine, Theophylline

## Abstract

**Background:**

Caffeine is one of the most abundant methylxanthines in tea, and it remains stable in processing of general teas. In the secondary metabolism of microorganism, theophylline is the main conversion product in caffeine catabolism through demethylation. Microorganisms, involved in the solid-state fermentation of pu-erh tea, have a certain impact on caffeine level. Inoculating an appropriate starter strain that is able to convert caffeine to theophylline would be an alternative way to obtain theophylline in tea. The purpose of this study was to isolate and identify the effective strain converting caffeine to theophylline in pu-erh tea, and discuss the optimal conditions for theophylline production.

**Results:**

Caffeine content was decreased significantly (*p* < 0.05) and theophylline content was increased significantly (*p* < 0.05) during the aerobic fermentation of pu-erh tea. Five dominant fungi were isolated from the aerobic fermentation and identified as *Aspergillus niger*, *Aspergillus sydowii*, *Aspergillus pallidofulvus*, *Aspergillus sesamicola* and *Penicillium mangini*, respectively. Especially, *A. pallidofulvus*, *A. sesamicola* and *P. mangini* were detected in pu-erh tea for the first time. All isolates except *A. sydowii* TET-2, enhanced caffeine content and had no significant influence on theophylline content. In the aerobic fermentation of *A. sydowii* TET-2, 28.8 mg/g of caffeine was degraded, 93.18% of degraded caffeine was converted to theophylline, and 24.60 mg/g of theophylline was produced. *A. sydowii* PET-2 could convert caffeine to theophylline significantly, and had application potential in the production of theophylline. The optimum conditions of theophylline production in the aerobic fermentation were 1) initial moisture content of 35% (w/w), 2) inoculation quantity of 8%, and 3) incubation temperature at 35 °C.

**Conclusions:**

For the first time, we find that *A. sydowii* PET-2 could convert caffeine to theophylline, and has the potential value in theophylline production through aerobic fermentation.

## Background

Methylxanthines are natural and synthetic compounds found in many foods, drinks, pharmaceuticals, and cosmetics [1]. Caffeine (1, 3, 7-trimethylxanthine or 3, 7-dihydro-1, 3, 7-trimethyl-1H-2, 6dione) is one of the most abundant methylxanthines in tea, it counts 30–50 mg/g of dry matter weight. Several bacteria and fungi have been selected from the soil of tea and coffee plantations to degrade caffeine. The bacteria which have capability of degrading caffeine are mainly including *Pseudomonas putida* [2, 3], *Pseudomonas lutea* [4] and *Serratia marcescens* [5]*.* Caffeine-degrading fungi [6, 7] are *Aspergillus tamarii*, *Fusarium solani*, *Aspergillus niger* and *Penicillium commune.* In the secondary metabolism of microorganisms, both demethylation and oxidation are found in caffeine degradation, and demethylation is the main caffeine degradation pathway [2]. However, under the effect of caffeine-degrading fungi, theophylline (1, 3-dimethyxanthine) is the major metabolite of caffeine [3], and it is different from the metabolites in bacteria [8].

Caffeine level remains stable in the processing of general teas (green tea, black tea, oolong tea and white tea) [9, 10]. Tea cultivar is the only factor could impact caffeine content in fresh tea leaves [11]. Pu-erh tea (pu-erh shucha) is produced through a natural solid-state fermentation process by using the sun-dried green tea leaves (*Camellia sinensis var. assamica* (JW Masters) Kitamura) as the raw material [12–14]. Caffeine content is changing in pu-erh tea, which should be attributed to the participation of microorganisms found in the solid-state fermentation [15–18]. Some reports found that several microorganisms such as *Candida albicans, Candida famata* (*Debaryomyces hansenii*)*, Aspergillus niger* and *Aspergillus sydowii* have potential capability to reduce caffeine content through an inoculated fermentation [18–20]. This suggests that the effective strain with the capability of converting caffeine to theophylline could be found in pu-erh tea.

Theophylline is a common prescribed drug for treatment of asthma and chronic obstructive pulmonary disease (COPD) through bronchodilator and anti-inflammatory effects on the respiratory system [21]. In addition, theophylline can be used as an initial treatment in asthma attacks and other clinical conditions, together with β2-mimetic drugs and corticosteroids [22]. During the caffeine catabolism of tea tree (*Camellia sinesis* (L.) O. Kuntze), theophylline is a transient metabolite and stays at a very low level as one of natural methylxanthines [23]. Hence, inoculation of an effective strain/ effective strains during the fermentation of tea leaves is an alternative way to produce theophylline.

In this paper, five fungal strains were isolated from the aerobic fermentation of pu-erh tea and identified as *A. niger*, *A. sydowii*, *Aspergillus pallidofulvus*, *Aspergillus sesamicola* and *Penicillium mangini*, respectively. *A. sydowii* PET-2 could convert caffeine to theophylline, which had a potential value in theophylline production. In addition, the optimal conditions for caffeine degradation and theophylline production were discussed.

## Results

### Differences between aerobic and anaerobic fermentation

**Fig. 1:**
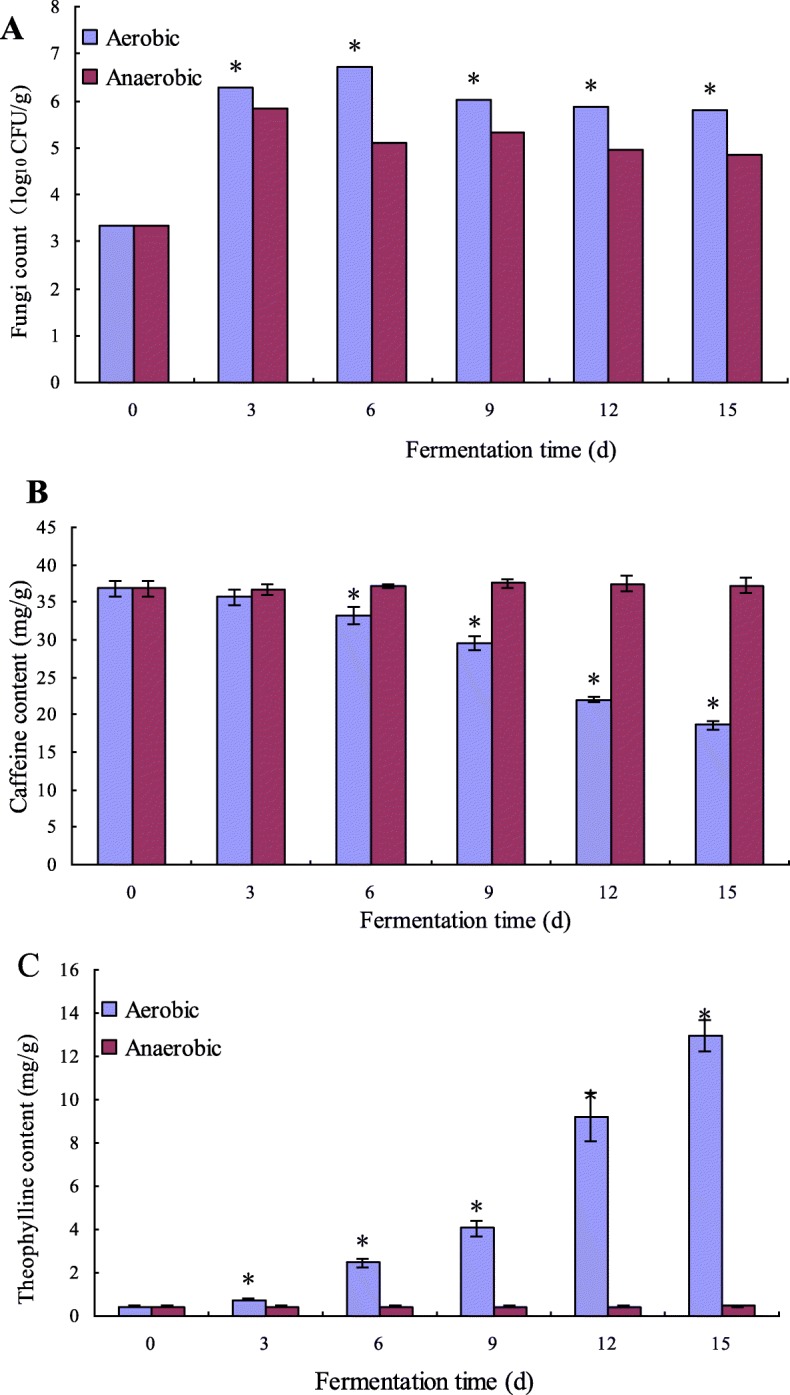
Differences between aerobic and anaerobic fermentation in fungi count (**a**), caffeine (**b**) and theophylline (**c**).Data are presented as mean value ± SD of three independent tests. * indicated there is significant difference between aerobic and anaerobic fermentation (Duncan’s multiple range test: p < 0.05)

In a previous study [20], caffeine content was decreased significantly (*p* < 0.05) during the solid-state fermentation of pu-erh tea. Aerobic and anaerobic fermentation was carried out at a natural condition to simulate the solid-state fermentation of pu-erh tea. Fungi count, caffeine and theophylline contents were determined in the fermentation. Results are presented in Fig. 1. There were significant (*p* < 0.05) differences in fungi count, caffeine and theophylline contents between aerobic and anaerobic fermentation. Compared with the anaerobic fermentation, caffeine content was decreased significantly (*p* < 0.05) in the aerobic fermentation; about 16.73 mg/g of caffeine of dry matter weight of tea leaves was degraded at the end of the fermentation. Furthermore, theophylline content was increased significantly (*p* < 0.05) along with caffeine degradation and about 15.52 mg/g of theophylline was produced at the end of the fermentation. As shown in Fig. 1A, fungal colony count increased dramatically from day 0 to day 6 and maintained at a high level during the whole fermentation. The microbial metabolism in aerobic fermentation was more active than anaerobic fermentation. Based on the changes of caffeine and theophylline contents, we predicted that the fungi in the aerobic fermentation lead to the conversion of caffeine to theophylline. Therefore, samples collected from the aerobic fermentation were used to isolate potential starter strains and the dominant fungi were analyzed in this paper.

### Identification results of fungi in aerobic fermentation

**Fig. 2:**
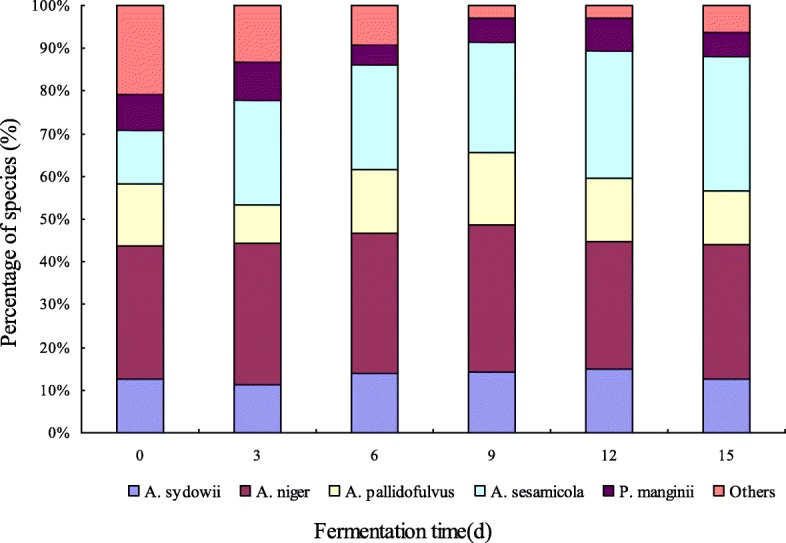
Changes in distribution of dominant fungi during pu-erh tea aerobic fermentation

A total of 5 fungal strains were isolated from the aerobic fermentation of pu-erh tea. The received sequence (546 bp) of PET-1 is provided in Additional file 1: Fig. S1. The received sequence (516 bp) of PET-2 is provided in Additional file 1: Fig. S2. The received sequences (541 bp ITS sequence, 516 bp β-Tubulin sequence and 765 bp Calmodulin sequence, respectively) of PET-3 are provided in Additional file 1: Fig. S3. The received sequences (532 bp ITS sequence, 515 bp β-Tubulin sequence and 757 bp Calmodulin sequence, respectively) of PET-4 are provided in Additional file 1: Fig. S4. The received sequences (525 bp ITS sequence and 420 bp β-Tubulin sequence, respectively) of PET-5 are provided in Additional file 1: Fig. S5. After the molecular identification, 5 fungal strains were identified as *A. niger* (99.8% sequence identity with strain NCBT110A), *A. sydowii* (99.8% sequence identity with strain NRRL250), *A. pallidofulvus* (99.9% sequence identity with strain NRRL4789), *A. sesamicola* (99.8% sequence identity with strain CBS137324) and *P. mangini* (99.6% sequence identity with strain CBS253.31), respectively (Table 1). As shown in Fig. 2, all fungal strains were detected at various stages in the aerobic fermentation. The isolated fungi account for 86.67–97.14% in total colony count from day 3 to day 15, which could be considered as the dominant fungi in the fermentation.

**Table 1:** Dominant fungi isolated from the aerobic fermentation of pu-erh tea and identified by sequence determination

Strains	gene	Primers	Fragments (bp)	Species	Strain number	identity
PET-1	ITS	ITS1/ITS4	546	*Aspergillus niger*	NCBT110A	99.8%
PET-2	ITS	ITS1/ITS4	516	*Aspergillus sydowii*	NRRL250	99.8%
PET-3	ITS	TS1/ITS4	541	*Aspergillus pallidofulvus*	NRRL4789	99.9%
	β-Tubulin	Bt2a/Bt2b	516			
	Calmodulin	CF1L/CF4	765			
PET-4	ITS	TS1/ITS4	532	*Aspergillus sesamicola*	CBS 137324	99.8%
	β-Tubulin	Bt2a/Bt2b	515			
	Calmodulin	CF1L/CF4	757			
PET-5	ITS	TS1/ITS4	525	*Penicillium manginii*	CBS253.31	99.6%
	β-Tubulin	Bt2a/Bt2b	420

### Effects of microorganisms on the conversion of caffeine to theophylline

**Fig. 3:**
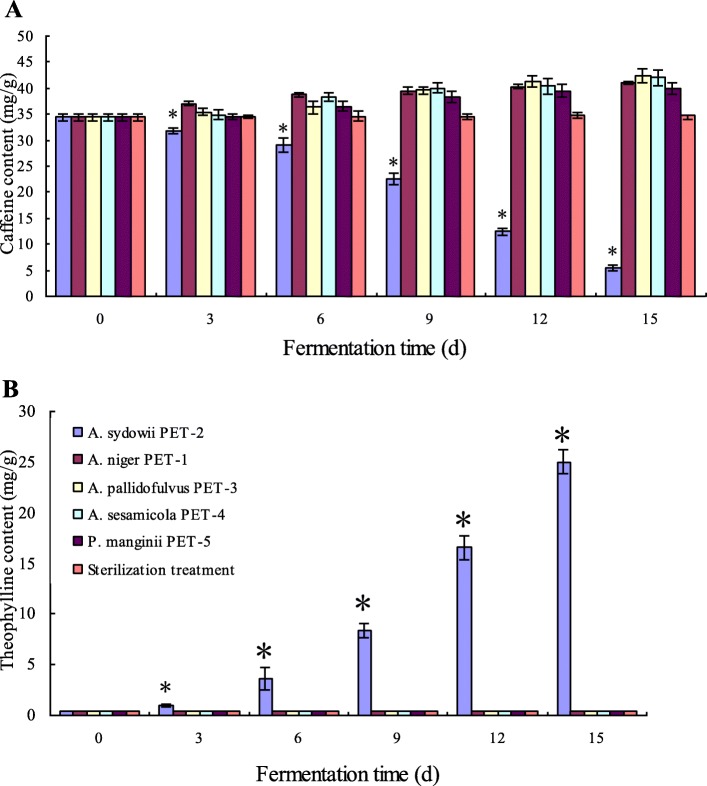
Changes in contents of caffeine (**a**) and theophylline (**b**) in aerobic fermentation inoculated by tea-derived strains, and sterilization treatment group. Data are presented as mean value ± SD of three replications. * indicated there is significant difference (*p* < 0.05) between *A. sydowii* PET-2 fermentation group and other treatment groups according to Duncan’s test

In order to find out the effects of microorganisms on the conversion of caffeine to theophylline, the isolated fungal strains were inoculated into the sun-dried tea leaves and cultivated in the incubator at 30 °C for the aerobic fermentation, respectively. As shown in Fig. 3, different microorganisms affected on caffeine and theophylline contents differently. Compared with the sterilization treatment, *A. niger* PET-1, *A. pallidofulvus* PET-3, *A. sesamicola* PET-4 and *P. mangini* PET-5 increased caffeine content significantly (Fig. 3a), which should be attributed to microbial metabolism and nutrient loss. However, there was no significant increase of theophylline content in the aerobic fermentation of *A. niger* PET-1, *A. pallidofulvus* PET-3, *A. sesamicola* PET-4 and *P. mangini* PET-5, respectively (Fig. 3b). During the fermentation inoculated by *A. sydowii* PET-2, caffeine content was decreased significantly (*p* < 0.05) and theophylline content was increased significantly (*p* < 0.05) (Fig. 3). At the end of the fermentation (day 15), 28.85 mg/g of caffeine was degraded with an amplitude of about 86.80%. With the degrading of caffeine, 24.60 mg/g of theophylline was produced at day 15 and became the main methylxanthine in the aerobic fermentation. Therefore, *A. sydowii* PET-2 had the capability of converting caffeine to theophylline,

### Ability of *A. sydowii* PET-2 converting caffeine to theophylline

*A. sydowii* PET-2 was inoculated into the sun-dried tea leaves for the aerobic fermentation and the kinetic parameters were established to study the conversion of caffeine to theophylline. As the fermentation progressed, tea polyphenols, and the main tea pigments including theaflavins, thearubigins and theabrownins contents were measured. The results are provided in Additional file 2:Table S1. During the aerobic fermentation, tea polyphenols content was decreased significantly (*p* < 0.05), theabrownins content was increased significantly (*p* < 0.05). Similar changes of tea polyphenols and theabrownins contents were found in the solid-sate fermentation of pu-erh tea. In the end of the fermentation, theabrownins content reached the national quality requirements of pu-erh tea. Therefore, *A. sydowii* PET-2 could be used in the pu-erh tea fermentation.

The kinetic parameters in the inoculated fermentation, including caffeine content (*C*_caffeine,t_), theophylline content (*C*_theophylline,t_), the yield of theophylline (Y_theophylline/caffeine_), caffeine removal ratio (%) and theophylline production (mg/g) are listed in Table 2. As shown in Table 2, approximately 7.25, 15.52, 34.47, 63.93, and 83.88% of caffeine were degraded at day 3, 6, 9, 12 and 15, respectively. Moreover, theophylline was detected and produced with the degrading of caffeine. At the end of the fermentation, 24.60 ± 1.18 mg/g of theophylline was produced, the yield of theophylline stayed at 85%, and about 93.18% of degraded caffeine was converted to theophylline. The production of theophylline was consistent with the tendency of caffeine degradation. *A. sydowii* PET-2 could lead caffeine degradation and convert most of the degraded caffeine to theophylline in the inoculated fermentation, which indicated that *A. sydowii* PET-2 had the most capability of converting caffeine to theophylline.

**Table 2:** Kinetic parameters for the conversion of caffeine to theophylline in the fermentation of *A. sydowii* PET-2

Time (d)	*C*_caffeine,t_ (mg/g)	*C*_theophylline,t_ (mg/g)	Y theophylline/caffeine	Caffeine removal ratio (%)	Theophylline production (mg/g)
0	34.39 ± 0.69	0.43 ± 0.04	ND	ND	ND
3	31.89 ± 0.51	0.98 ± 0.20	0.23 ± 0.09	7.25 ± 2.82	0.55 ± 0.19
6	29.05 ± 1.45	3.62 ± 1.10	0.59 ± 0.10	15.52 ± 4.11	3.19 ± 1.08
9	22.54 ± 1.00	8.39 ± 0.72	0.67 ± 0.08	34.47 ± 2.30	7.96 ± 0.69
12	12.40 ± 0.76	16.54 ± 1.16	0.73 ± 0.06	63.93 ± 2.77	16.11 ± 1.15
15	5.54 ± 0.55	25.03 ± 1.17	0.85 ± 0.03	83.88 ± 1.77	24.60 ± 1.18

All data are presented as mean value ± SD of three replications, ND was not detected.

Contents of caffeine and theophylline were determined by HPLC.

Samples collected on 0 day were the raw material for the fermentation.

*C*_caffeine,t_ was the caffeine content (mg/g) detected in the fermentation; *C*_theophylline,t_ was the theophylline content (mg/g) detected in the fermentation;Y_theophylline/caffeine_ = theophyline yield on caffeine (mg/mg). Caffeine removal ratio (%) = (*C*_caffeine,0_-C_caffeine,t_)/C_caffeine,0_*100%; Theophylline production (mg/g) = *C*_theophylline,t_-*C*_theophylline,0_.

Due to the caffeine conversion characteristic, *A. sydowii* PET-2 had broad application prospects in the production of theophylline via an aerobic fermentation. The influence factors of theophylline production such as moisture content, inoculation quantity and incubation temperature were discussed later in this paper.

### Selection of optimal condition in moisture content with inoculation quantity of 4% and incubation temperature at 30 °C

**Fig. 4:**
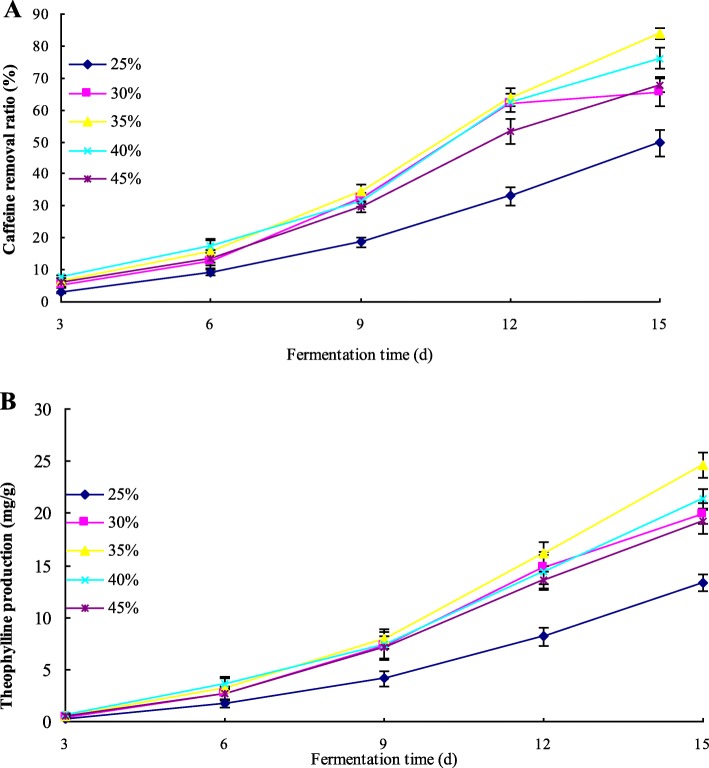
Effect of moisture content in an aerobic fermentation inoculated by *A. sydowii* PET-2 on caffeine degradation (**a**) and theophylline production (**b**).Data are presented as mean value ± SD of three replications. Caffeine removal ratio = (C_caffeine,0_-C_caffeine,t_)/C_caffeine,0_*100%; Theophylline production = C_theophylline,t_-C_theophylline,0_.C_caffeine,0_ was the initial caffeine content (mg/g), C_caffeine,t_ was the caffeine content (mg/g) detected in the fermentation. C_theophylline,t_ was the theophylline content (mg/g) detected in the fermentation, C_theophylline,0_ was the initial theophylline content (mg/g)

*A. sydowii* PET-2 was inoculated into the sun-dried tea leaves with increasing initial moisture contents (25, 30, 35, 40 and 45%, respectively) and cultivated in the incubator at the temperature of 30 °C. Caffeine and theophylline contents were determined by HPLC during the fermentation. Caffeine removal ratio (%) and theophylline production (mg/g) were calculated to investigate the influence of moisture content on caffeine conversion. As shown in Fig. 4, caffeine removal ratio and theophylline production increased steadily with the fermentation. The microbial metabolism was influenced by moisture content, thus moisture content had significant impacts on caffeine degradation and theophylline production. The experiments indicated that the fermentation group with the initial moisture content of 35% (w/w) had the highest caffeine removal ratio and theophylline production. In addition, caffeine removal ratio and theophylline production decreased significantly (*p* < 0.05) in lower moisture content. Only about 49.78% of caffeine was degraded and 13.34 mg/g of theophylline was produced in 25% (w/w) of moisture content at day 15. The single factor analysis showed that 35% (w/w) was the appropriate initial moisture content for the conversion of caffeine to theophylline.

### Selection of optimal condition in inoculation quantity with initial moisture content of 35% (w/w) and incubation temperature at 30 °C

**Fig. 5:**
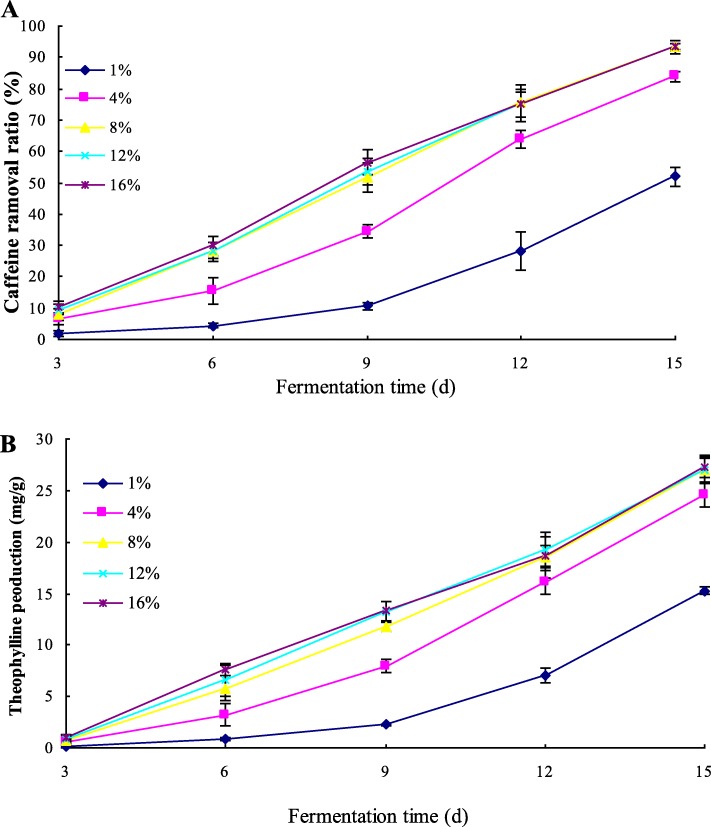
Effect of inoculation level in an aerobic fermentation inoculated by *A. sydowii* PET-2 on caffeine degradation (**a**) and theophylline production (**b**). Data are presented as mean value ± SD of three replications.Caffeine removal ratio = (C_caffeine,0_-C_caffeine,t_)/C_caffeine,0_*100%; Theophylline production = C_theophylline,t_-C_theophylline,0_.C_caffeine,0_ was the initial caffeine content (mg/g), C_caffeine,t_ was the caffeine content (mg/g) detected in the fermentation. C_theophylline,t_ was the theophylline content (mg/g) detected in the fermentation, C_theophylline,0_ was the initial theophylline content (mg/g)

A set of different inoculation quantities was built to find out the optimal inoculation quantity for caffeine conversion. Caffeine removal ratio (%) and theophylline production (mg/g) were calculated and showed in Fig. 5. In low inoculation quantity (1%), the caffeine conversion was inhibited, only about 51.54% of caffeine was degraded and 15.72 mg/g of theophylline was produced at day 15. With the increasing inoculation quantity applied (from 1 to 8%), the caffeine conversion capability increased obviously. However, in high inoculation level (8, 12, and 16%, respectively), there were no significant (*p* > 0.05) changes in caffeine removal ratio and theophylline production. Through comparison and analysis, 8% was the optimal inoculation quantity with the higher caffeine removal ratio and theophylline production.

### Selection of optimal condition in incubation temperature with initial moisture of 35% (w/w) and inoculation quantity of 4%

**Fig. 6:**
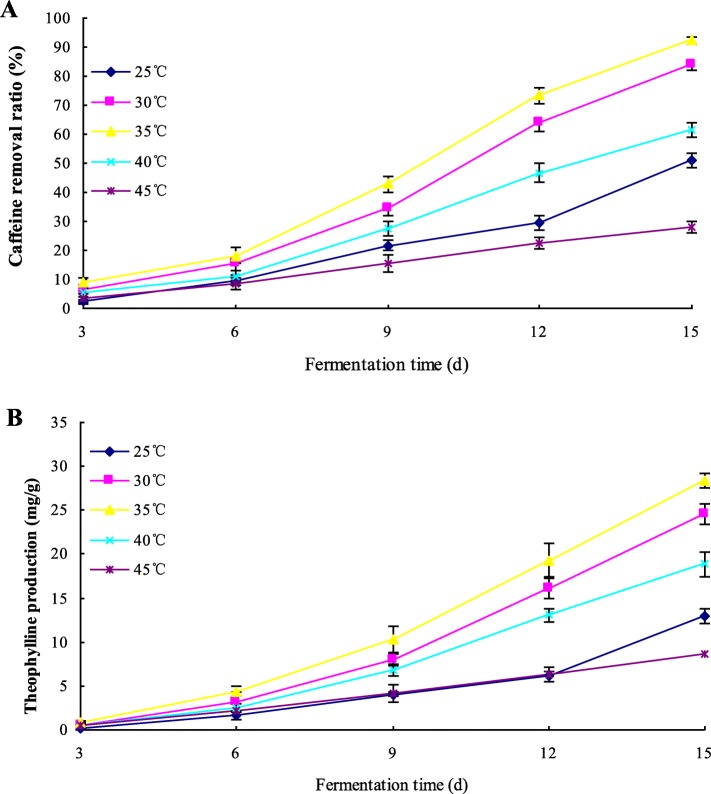
Effect of incubation temperature in an aerobic fermentation inoculated by *A. sydowii* PET-2 on caffeine degradation (**a**) and theophylline production (**b**). Data are presented as mean value ± SD of three replications.Caffeine removal ratio = (C_caffeine,0_-C_caffeine,t_)/C_caffeine,0_*100%; Theophylline production = C_theophylline,t_-C_theophylline,0_.C_caffeine,0_ was the initial caffeine content (mg/g), C_caffeine,t_ was the caffeine content (mg/g) detected in the fermentation. C_theophylline,t_ was the theophylline content (mg/g) detected in the fermentation, C_theophylline,0_ was the initial theophylline content (mg/g)

Incubation temperature had a significant impact on microbial metabolism. In order to compare the caffeine conversion level from caffeine to theophylline in different incubation temperatures, *A. sydowii* PET-2 was inoculated into the sun-dried tea leaves with an initial moisture content of 35% (w/w) and an inoculation quantity of 4%, and cultivated in the incubator with different incubation temperatures (25, 30, 35, 40 and 45 °C, respectively) for 15 days. Caffeine and theophylline contents were determined, and caffeine removal ratio (%) and theophylline production (mg/g) are showed in Fig. 6. In the temperature range between 25 and 35 °C, caffeine removal ratio and theophylline production increased significantly (*p* < 0.05). Between 40 and 45 °C, because the growth and metabolism of microorganism were inhibited, caffeine removal ratio and theophylline production declined obviously. Therefore, 35 °C was the optimal temperature for the conversion of caffeine to theophylline.

## Discussion

Pu-erh tea is one of the traditional Chinese teas. Based on the recent researches, pu-erh tea has a certain positive effect on human health, including reducing waist fat, increasing antioxidant capacity and anti-inflammatory ability, and preventing atherosclerosis and cancer [24–26]. Due to the benefits in antidiabetics, hypolipidemic and antirheumatic [27–29], pu-erh tea has been produced and drank by the minority groups of the Yunnan people in China for centuries [30].

Solid-state fermentation is the key process in pu-erh tea, which includes aerobic fermentation in surface and anaerobic fermentation in centre [31]. The species within the genera *Aspergillus*, *Penicillium*, *Rhizomucor*, *Mucor*, *Cladosporium* and *Eurotium* are reported widely in pu-erh tea solid-state fermentation [32, 33]. *Aspergillus* spp. and *Saccharomyces* spp. were the main fungi in aerobic and anaerobic fermentation of pu-erh tea [30, 34]. *A. niger, A. tamarii, Aspergillus tubingensis, Aspergillus fumigatus, Aspergillus acidus, Aspergillus awamori, Blastobotrys adeninivorans, Candida tropicalis, Fusarium graminearum, Millerozyma* (syn. *Pichia*) *farinosa, Rasamsonia byssochlamydoides, Rasamsonia emersonii, Rasamsonia cylindrospora, Rhizomucor pusillus, Rhizomucor tauricus,* and *Thermomyces lanuginosus* have been detected in pu-erh tea [35–38]. In this study, 5 fungal strains were selected and identified as *A. niger*, *A. sydowii*, *A. pallidofulvus*, *A. sesamicola* and *P. mangini* from the aerobic fermentation of pu-erh tea, respectively.

Recently, *A. niger* has been reported widely and considered as the predominant fungi contributing to the fermentation and quality of pu-erh tea [31, 39, 40]. *A. niger* was detected in the aerobic fermentation of pu-erh tea and superior in the quantity, which conformed with the previous studies [32–34]. Other fungal species were rarely detected in pu-erh tea fermentation. Especially, *A. pallidofulvus*, *A. sesamicola* and *P. mangini* were found in pu-erh tea for the first time.

During the solid-state fermentation of pu-erh tea, fungi have significant impacts in chemical components and tea quality [41]. In the physiology of tea tree (*Camellia sinesis* (L.) O. Kuntze), theophylline is the transient metabolite in caffeine catabolism and stays at a very low level [23, 42]. Furthermore, the primarily metabolic pathway via caffeine-theophylline route in fungi has been reported and theophylline is the main conversion product in caffeine catabolism through demethylation [43, 44]. The influences of the isolated fungi on caffeine and theophylline metabolism were discussed in this paper. *A. niger* PET-1, *A. pallidofulvus* PET-3, *A. sesamicola* PET-4 and *P. mangini* PET-5 increased caffeine content significantly through the inoculated fermentation. In contrast, during the aerobic fermentation of *A. sydowii* PET-2, caffeine content was decreased significantly (*p* < 0.05). At the end of the fermentation, about 83.88% of caffeine (28.8 mg/g of caffeine) was degraded, 93.18% of degraded caffeine was converted to theophylline, and 24.60 ± 1.18 mg/g of theophylline was produced. Therefore, compared with other fungal strains, *A. sydowii* PET-2 was an effective strain converting caffeine to theophylline, which could be used in the production of theophylline through an aerobic fermentation.

Previous researches showed that *A. sydowii* has a high biotechnological potential [45, 46], it could produce monosaccharide [47] and indole alkaloids to restrain the proliferation of cells A549 [48]. The optimum conditions required for the production of theophylline such as moisture content, inoculation quantity and incubation temperature in aerobic fermentation were discussed in this study. The single factor analysis showed that the optimum conditions of theophylline production were 1) initial moisture content of 35% (w/w), 2) inoculation quantity of 8%, and 3) incubation temperature at 35 °C. The optimum conditions provided the relevant information for the conversion of caffeine to theophylline by *A. sydowii* PET-2.

## Conclusions

Five fungal strains were isolated from the aerobic fermentation of pu-erh tea in natural condition. Among them, *A. pallidofulvus*, *A. sesamicola* and *P. mangini* were detected in pu-erh tea for the first time. Moreover, *A. niger* PET-1, *A. pallidofulvus* PET-3, *A. sesamicola* PET-4 and *P. mangini* PET-5 could increase caffeine content and had no significant impact on theophylline content. *A. sydowii* PET-2 was an effective strain converting caffeine to theophylline, which could be used for theophylline production. In addition, the optimum conditions of theophylline production in *A. sydowii* PET-2 aerobic fermentation were identified in this study, which provides the suggestions for the application of *A. sydowii* in theophylline production.

## Methods

### Material and reagents

Sun-dried green tea leaves (*C. sinensis* var. *assamica*) with moisture content 6.25% by weight were obtained from Yunnan province, China. SP fungal DNA kit, DNA marker, polymerase chain reaction (PCR) spread reagent, primers: ITS1 (5`-TCCGTAGGTGAACCTGCGG-3`) and ITS4 (5`-TCCTCCGCTTATTGATAGC-3`); Bt2a (5`-GGTAACCAAATCGGTGCTGCTTTC-3`) and Bt2b (5`-ACCCTCAGTGTAGTGACCCTTGGC-3`); and CF1L (5`-GCTGACTCGTTGACCGAAGAG-3`) and CF4 (5`-ATTTTTGCATCATGAGCTGAAC-3`) were purchased from Japan TaKaRa company. Acetonitrile and acetic acid for HPLC were purchased from Beijing Mreda Biotechnology Company, China.

### Aerobic and anaerobic fermentation at a natural condition

The whole solid-state fermentation of pu-erh tea contains two phases: aerobic and anaerobic fermentation [31]. In this study, aerobic and anaerobic fermentation was carried out in Tea Processing Laboratory, College of Long Run Pu-erh Tea, Yunnan Agricultural University, Kunming of Yunnan province. About 400 g of sun-dried green tea leaves moistened with 220 mL distilled water to achieve an added moisture content of 35% (w/w) and stored loosely in a 1000-mL Bunsen beaker for the aerobic fermentation [39]. The leaves were turned over before the collection of samples. In addition, another 400 g of sun-dried green tea leaves mixed with 220 mL distilled water were divided into six groups and each of them about 100–110 g was stored tightly in 150-mL glass bottles with about 15 mL head-space. The bottles were completely sealed by lid and covered with gas-tight polyethylene film for the anaerobic fermentation. The fermentation was carried out at room temperature (25 ± 2 °C) based on the natural microbiota existing on the leaves [36]. The whole fermentation process continued for 15 days at a nature condition.

Tea samples from aerobic and anaerobic fermentation were collected at day 0, 3, 6, 9, 12, 15, respectively. The samples were divided into two parts. One part was stored at 4 °C for further characterization and identification of fungi. Another part was dried at 80 °C for 4 h for chemical components analysis. In the aerobic fermentation, caffeine content was declined significantly (*p* < 0.05) and theophylline content was increased significantly (*p* < 0.05). The samples from the aerobic fermentation were furthermore chose for the isolation and identification of the effective strain converting caffeine to theophylline.

### Isolation and calculation of fungi

Fermented samples would be used to isolate the fungi using potato dextrose agar (PDA) medium and they were counted by dilution-plating method [35]. The colony forming units (CFU) was calculated by per gram of sun-dried tea leaves after 2 days of cultivation at 30 °C.

### Fungal identification

The target strains grew aerobically as pure cultures in 20 mL of Czapek Dox medium in 125 mL shake flasks at 30 °C, 250 rpm, for 2 d. The fresh cells were obtained by centrifugation at 1700 g for 5 min and freeze-dried at − 80 °C [39, 40]. DNA was extracted by using SP fungal DNA kit. The fungal primers ITS1 and ITS4, Bt2a and Bt2b, and CF1L and CF4 were used in the PCR to amplify the ITS, β-tubulin and Calmodulin regions, respectively [31, 32, 39]. The final volume of 50 μL, 1.0 *μ*L of containing template DNA, 5 *μ*L of 10 x buffer, 5 *μ*L of dNTPs (2.5 mM), 0.5 *μ*L of Taq polymerase, 1.0 *μ*L (10 μM) of each primer, and 36.5 *μ*L of sterile distilled water were used to implement amplifications [31, 39]. The PCR reaction procedure was as follows. Pre-degeneration at 95 °C for 5 min, degeneration at 94 °C for 1 min, annealing at 54 °C for 1 min, extension at 72 °C for 1.5 min, with 35 cycles, extension at 72 °C for 10 min [33]. It was stored at 10 °C in the end of the reaction process.

The PCR was produced in an ABI3730 automatic DNA sequencer (Applied Biosystems, USA) [20]. The received sequences were sent to Genbank of NCBI to seek similar sequences of type strain by using Blastn [35]. Multiple sequence alignment was carried out by using Clustal X for Windows. The evolution distance was calculated through a Kimura2-parameter of the MEGA 4.0 Soft.

### Aerobic fermentation inoculated by microbial isolate

400 g of sun-dried green tea leaves mixed with moderate distilled water was divided into six groups and each of them about 100–110 g was stored in a 150-mL gas-permeable Tissue culture vessel for the aerobic fermentation [39]. Before the inoculation, high temperature sterilization at 121 °C for 5 min was carried out to kill viable microorganisms with minimal damage in main functional components [20, 39].

Two loopfuls of isolated strains were transferred aseptically from a dish slant into 25 mL of sterile tea infusion in a 125 mL Erlenmeyer flask [20]. The flask was incubated aerobically at 30 °C for 48 h on the incubator shaker (250 rpm) [39]. The cell density was adjusted to 1.0 × 10^7^ CFU/mL and the inoculum was stored at 4 °C for the inoculation. About 4 mL above noted inoculum was added into per 100 g of dry weight sterile sun-dried tea leaves to obtain an inoculation quantity of 4%. The initial moisture content was 35% (w/w). Sterilization treatment with the identical moisture content was carried out as the control group. The culture vessels were incubated in a constant temperature incubator at 30 °C. Tea samples of inoculated fermentation and sterilization treatment were collected at day 0, 3, 6, 9, 12, 15, respectively. Caffeine and theophylline contents were determined by HPLC.

### Optimization in aerobic fermentation of target strain

Experiments were performed during 15 days in an aerobic solid-state fermentation inoculated by target strain to evaluate the conversion level of caffeine to theophylline. Effects of moisture content, inoculation quantity and incubation temperature on caffeine degradation and theophylline production were analyzed [45].

1. Moisture content. A set of different moisture contents (25, 30, 35, 40, and 45% w/w, respectively) were built with given inoculation quantity of 4%. The inoculated leaves were incubated in a constant temperature incubator at 30 °C for 0, 3, 6, 9, 12 and 15 days, respectively.
2. Inoculation quantity. A set of different inoculation quantities (1, 4, 8, 12, and 16%, respectively) were built and inoculated into the mixed tea to reach the identical moisture content of 35% (w/w). The inoculated leaves were incubated in a constant temperature incubator at 30 °C for 0, 3, 6, 9, 12 and 15 days, respectively.
3. Incubation temperature. Sun-dried green tea leaves were moistened with distilled water to achieve the given moisture content of 35% (w/w). 4% of above noted inoculum was inoculated into the sterile tea leaves. The culture vessels were incubated in a constant temperature incubator at different incubation temperatures (25, 30, 35, 40, and 45 °C, respectively). Tea samples were collected at day 0, 3, 6, 9, 12, 15, respectively.

Caffeine and theophylline contents were determined by HPLC.

### Determination of caffeine and theophylline by HPLC

In order to determine caffeine and theophylline contents, the tea samples were extracted by boiling water for 45 min. Caffeine and theophylline contents were determined by Agilent 1200 HPLC equipment using Agilent C_18_ Chromatogram column (250 mm × 4.6 mm, 5 μm) with solvent A (100% acetonitrile) and solvent B (0.2% v/v acetic acid water solution) as mobile phase [49]. The gradient was programmed as follows. The mobile phase (at 0 min) consisted of 92% (v/v) solvent A (100% acetonitrile) and 8% (v/v) solvent B (0.2% v/v acetic acid water solution). Solvent A was then decreased linearly to 69% (v/v) at 50 min, whereas solvent B was increased linearly to 31% (v/v) at 50 min. The flow rate was 1.0 mL/min and 10 μL of extraction was injected. The column temperature set at 30 °C and the monitored wavelength was 280 nm.

### Statistical analysis

Caffeine removal ratio, theophylline production, Y_theophylline/caffeine_ and conversion ratio were used to evaluate the capability of target strain conversing caffeine to theophylline.

Caffeine removal ratio (%) was estimated as follow:

1 $$ \mathrm{Caffeine}\ \mathrm{removal}\ \mathrm{ratio}=\left({\mathrm{C}}_{\mathrm{caffeine},0}-{\mathrm{C}}_{\mathrm{caffeine},\mathrm{t}}\right)/{\mathrm{C}}_{\mathrm{caffeine},0}\ast 100\% $$

Theophylline production (mg/g) was estimated as follow:

2 $$ \mathrm{Theophylline}\ \mathrm{production}={\mathrm{C}}_{\mathrm{theophylline},\mathrm{t}}-{\mathrm{C}}_{\mathrm{theophylline},0} $$

Y_theophylline/caffeine_ was the yield of theophylline on caffeine and estimated as follow:

3 $$ {\mathrm{Y}}_{\mathrm{theophylline}/\mathrm{caffeine}}=\left({\mathrm{C}}_{\mathrm{theophylline},\mathrm{t}}-{\mathrm{C}}_{\mathrm{theophylline},0}\right)/\left({\mathrm{C}}_{\mathrm{caffeine},0}-{\mathrm{C}}_{\mathrm{caffeine},\mathrm{t}}\right) $$

Conversion ratio (%) was used to evaluate the conversion efficiency from caffeine to theophylline and estimated as follow:

4 $$ \mathrm{Conversion}\ \mathrm{ratio}=\left[\left({\mathrm{C}}_{\mathrm{theophylline},\mathrm{t}}-{\mathrm{C}}_{\mathrm{theophylline},0}\right)/{\mathrm{M}}_{\mathrm{theophylline}}\right]/\left[\left({\mathrm{C}}_{\mathrm{caffeine},0}-{\mathrm{C}}_{\mathrm{caffeine},\mathrm{t}}\right)/{\mathrm{M}}_{\mathrm{caffeine}}\right]\ast 100\% $$

In Eqs. (1), (3) and (4), C_caffeine,0_ was the initial caffeine content (mg/g), C_caffeine, t_ was the caffeine content (mg/g) detected in the fermentation.

In Eqs. (2), (3) and (4), C_theophylline, t_ was the theophylline content (mg/g) detected in the fermentation, C_theophylline, 0_ was the initial theophylline content (mg/g).

In Eq. (4), M_theophylline_ was the molar mass of theophylline and M_caffeine_ was the molar mass of caffeine. In this paper, M_theophylline_ = 180.16 g/mol, M_caffeine_ = 194.19 g/mol, respectively.

Due to the uncertainty of the natural conditions, the natural fermentation was one parallel experiment. But the fungi count, caffeine and theophylline contents in the natural fermentation were three independent tests to ensure the credibility. The inoculated fermentation and optimization in aerobic fermentation were three replications to obtain reliable results. The mean value and standard deviation (SD) of analytic was calculated using SPSS 20.0 for Windows. The significant differences (*p* < 0.05) was analyzed using one-way analysis of variance (one-way ANOVA) by Duncan’s multiple-range test.

## Supplementary information

**Additional file 1 Figure S1.** The received sequence of strain PET-1. **Figure S2.** The received sequence of strain PET-2. **Figure S3.** The received sequences of strain PET-3. **Figure S4.** The received sequences of strain PET-4. **Figure S5.** The received sequences of strain PET-5.

**Additional file 2 **T**able S1.** Changes of other main chemical compounds in the fermentation of *A. sydowii* PET-2.

## Data Availability

The data that support the findings of this study are available from the corresponding author upon reasonable request.

## Abbreviations

ANOVA: Analysis of variance
CFU: Colony forming units
COPD: Chronic obstructive pulmonary disease
dNTPs: deoxy-ribonucleoside triphosphates
HPLC: High performance liquid chromatography
ITS: Internal transcribed spacer
MEGA: Molecular evolutionary genetics analysis
NCBI: National Center for Biotechnology Information
PCR: Polymerase chain reaction
PDA: Potato dextrose agar
PET: Pu-erh tea
SD: Standard deviation
SPSS: Statistical product and service solutions
