# Supplementary material for: Production of theophylline via aerobic fermentation of pu-erh tea using tea-derived fungi
Source: BMC Microbiol. 2019 Nov 26;19:261. doi: 10.1186/s12866-019-1640-2 (PMC6878699; doi:10.1186/s12866-019-1640-2)
Supplement: Supplementary file 1 — Additional file 1 Figure S1. The received sequence of strain PET-1. Figure S2. The received sequence of strain PET-2. Figure S3. The received sequences of strain PET-3. Figure S4. The received sequences of strain PET-4. Figure S5. The received sequences of strain PET-5. [file 12866_2019_1640_MOESM1_ESM.doc]

§

ITS Sequence of strain PET-1 (546bp):

1 TCTTTGGGCCCACCTCCCATCCGTGTCTATTATACCCTGTTGCTTCGGCG 50

51 GGCCCGCCGCTTGTCGGCCGCCGGGGGGGCGCCTTTGCCCCCCGGGCCCG 100

101 TGCCCGCCGGAGACCCCAACACGAACACTGTCTGAAAGCGTGCAGTCTGA 150

151 GTTGATTGAATGCAATCAGTTAAAACTTTCAACAATGGATCTCTTGGTTC 200

201 CGGCATCGATGAAGAACGCAGCGAAATGCGATAACTAATGTGAATTGCAG 250

251 AATTCAGTGAATCATCGAGTCTTTGAACGCACATTGCGCCCCCTGGTATT 300

301 CCGGGGGGCATGCCTGTCCGAGCGTCATTGCTGCCCTCAAGCCCGGCTTG 350

351 TGTGTTGGGTCGCCGTCCCCCTCTCCGGGGGGACGGGCCCGAAAGGCAGC 400

401 GGCGGCACCGCGTCCGATCCTCGAGCGTATGGGGCTTTGTCACATGCTCT 450

451 GTAGGATTGGCCGGCGCCTGCCGACGTTTTCCAACCATTTTTTCCAGGTT 500

501 GACCTCGGATCAGGTAGGGATACCCGCTGAACTTAAGCATATCAAA 546

Additional File 1: Figure S1 The received sequence of strain PET-1

ITS Sequence of strain PET-2 (516bp):

1 CTGCCTCCGGGCGCCCACCTCCCACCCGTGAATACCTAACACTGTTGCTT 50

51 CGGCGGGGAACCCCCTCGGGGGCGAGCCGCCGGGGACTACTGAACTTCA 100

101 TGCCTGAGAGTGATGCAGTCTGAGTCTGAATATAAAATCAGTCAAAACTTT 150

151 CAACAATGGATCTCTTGGTTCCGGCATCGATGAAGAACGCAGCGAACTGC 200

201 GATAAGTAATGTGAATTGCAGAATTCAGTGAATCATCGAGTCTTTGAACG 250

251 CACATTGCGCCCCCTGGCATTCCGGGGGGCATGCCTGTCCGAGCGTCATT 300

301 GCTGCCCATCAAGCCCGGCTTGTGTGTTGGGTCGTCGTCCCCCCCGGGGG 350

351 ACGGGCCCGAAAGGCAGCGGCGGCACCGTGTCCGGTCCTCGAGCGTATGG 400

401 GGCTTTGTCACCCGCTCGACTAGGGCCGGCCGGGCGCCAGCCGACGTCTC 450

451 CAACCATTTTTCTTCAGGTTGACCTCGGATCAGGTAGGGATACCCGCTGA 500

501 ACTTAAGCATATCAAA 516

Additional File 1: Figure S2 The received sequence of strain PET-2

ITS Sequence of strain PET-3 (541bp)：

1 TCGGGGCCACCTCCCACCCGTGTATACCGTACCTTGTTGCTTCGGCGAGC 50

51 CCGCCCCCTTTTTCTTAGGGGGGCACAGCGCTCGCCGGAGACACCAACGT 100

101 GAACACTGTCTGAAGTTTTGTCGTCTGAGTCGATTGTATCGCAATCAGTT 150

151 AAAACTTTCAACAATGGATCTCTTGGTTCCGGCATCGATGAAGAACGCAG 200

201 CGAAATGCGATAATTAATGTGAATTGCAGAATTCAGTGAATCATCGAGTC 250

251 TTTGAACGCACATTGCACCCCCTGGTATTCCGGGGGGTATGCCTGTCCGA 300

301 GCGTCATTGCTGCCCTCAAGCACGGCTTGTGTGTTGGGTCGTCGTCCCCC 350

351CCGGGGGACGGGCCCGAAAGGCAGCGGCGGCACCGCGTCCGGTCCTCGAG 400

401 CGTATGGGGCTTTGTCACACCGCTCTTGTAGGCCCGGCCGGCTGCTGGCC 450

451 GACGCTGAAAAGCAACCAACTATTTCTCCAGGTTGACCTCGGATCAGGTA 500

501 GGGATACCCGCTGAACTTAAGCATATCAATAAGCGGAGGAA 541

β-tubulin Sequence of strain PET-3 (516bp):

1 TCATCTTCGATACCTTGTGACCTATGACTCTCAATCCTTGATACTTGATA 50

51 CTTGTTTACTGATAGGTGAATAGGCAAAACATCTCTGGCGAGCACGGCCT 100

101 TGATGGCGCCGGTGTGTAAGTACATCCCGCGTTTACACCTATCGAAATCA 150

151 GAATCGACGAGAGAAGAAAAGAAAGAAATGATCATGGTGGGATTGATTGT 200

201 CTGATGGGATGAACAGTTACAATGGCTCCTCCGACCTTCAGCTGGAGCGC 250

251 ATGAACGTCTACTTCAACGAGGTTCGTTGCCCGAAAATTTTCTATCTCCT 300

301 TTCGCCGATCCGAAACGCCCCGTACAAGGCTCTAACCCACGCTTTCTTCA 350

351 TCTTCTAGGCTTCCGGTGGCAAGTATGTTCCCCGTGCCGTTCTGGTCGAT 400

401 CTTGAGCCCGGTACCATGGACGCTGTCCGTGCCGGTCCCTTCGGTCAGCT 450

451 TTTCCGCCCCGACAACTTCGTCTTCGGCCAGTCTGGTGCCGGTAACAACT 500

501 GGGCCAAGGGTCACTA 516

Calmodulin Sequence of strain PET-3 (765 bp):

1 TTCGTAGTGTTCCATTCTTCCTGATGTAATGATGGGGAAACGAAGCGATC 50

51 TTGCTTTGTTTTCTACGACTTCAGCCTTATGGGAATATTCCAAGCTCACA 100

101 TGAGATTTTGCCTCCTCACAGGACAAGGATGGCGATGGTTAGTGCTATTC 150

151 CGGTTTCCCTTCCAATTCATCGACTCACGCGACCGGCTATTTTCCGTCGA 200

201 TATGGAATTTTTTTCACCCTGTTGCTTCCGGCGACCGATTTGCGACCAGG 250

251 ACGCTAATTTGTGAATTCACGCTGCAGGCCAGATCACCACCAAGGAGTTG 300

301 GGCACTGTTATGCGCTCGCTGGGCCAGAACCCCTCCGAGTCTGAGTTGCA 350

351 GGACATGATCAACGAGGTTGACGCCGACAACAATGGCACCATCGATTTCC 400

401 CCGGTATGCGATGATGGACACACCGATATCTGGAAAGGGAGACAATCCTG 450

451 AAACTCAGCTGCTAACCTCACGCAGAGTTCCTGACGATGATGGCCCGAAA 500

501 GATGAAGGATACCGATTCTGAGGAGGAAATCCGGGAAGCTTTCAAGGTCT 550

551 TCGATCGCGATAACAACGGTTTCATCTCGGCCGCGGAGCTGCGCCACGTC 600

601 ATGACCTCCATCGGCGAGAAGCTCACCGACGACGAGGTCGATGAGATGAT 650

651 CCGTGAGGCGGACCAGGATGGCGACGGCCGGATTGACTGTACGTTGTGAC 700

701 CTTGGGATGCCCTTTCTGAACCCAAACTAATGAACTGCTCCAGACAACGA 750

751 GTTCGTCCAACTTAT 765

Additional File 1: Figure S3 The received sequences of strain PET-3

ITS Sequence of strain PET-4 (532bp)：

1 CCTCCCACCCGTGTATACCGTACCTTGTTGCTTCGGCGAGCCCGCCCCCT 50

51 TTTTCTTAGGGGGGCACAGCGCTCGCCGGAGACACCAACGTGAACACTGT 100

101 CTGAAGTTTTGTCGTCTGAGTCGATTGTATCGCAATCAGTTAAAACTTTC 150

151 AACAATGGATCTCTTGGTTCCGGCATCGATGAAGAACGCAGCGAAATGCG 200

201 ATAATTAATGTGAATTGCAGAATTCAGTGAATCATCGAGTCTTTGAACGC 250

251 ACATTGCACCCCCTGGTATTCCGGGGGGTATGCCTGTCCGAGCGTCATTG 300

301 CTGCCCTCAAGCACGGCTTGTGTGTTGGGTCGTCGTCCCCCCCGGGGGAC 350

351 GGGCCCGAAAGGCAGCGGCGGCACCGCGTCCGGTCCTCGAGCGTATGGGG 400

401 CTTTGTCACACCGCTCTTGTAGGCCCGGCCGGCTGCTGGCCGACGCTGAA 450

451 AAGCAACCAACTATTTCTCCAGGTTGACCTCGGATCAGGTAGGGATACCC 500

501 GCTGAACTTAAGCATATCAATAAGCGGAGGAA 532

β-tubulin Sequence of strain PET-4 (515bp):

1 CATCTTCGATACCTTGTGACCTATGACTCTCAATCCTTGATACTTGATAC 50

51 TTGTTTACTGATAGGTGAATAGGCAAAACATCTCTGGCGAGCACGGCCTT 100

101 GATGGCGCCGGTGTGTAAGTACATCCCGCGTTTACACCTATCGAAATCAG 150

151 AATCGACGAGAGAAGAAAAGAAAGAAATGATCATGGTGGGATTGATTGTC 200

201 TGATGGGATGAACAGTTACAATGGCTCCTCCGACCTTCAGCTGGAGCGCA 250

251 TGAACGTCTACTTCAACGAGGTTCGTTGCCCGAAAATTTTCTATCTCCTT 300

301 TCGCCGATCCGAAACGCCCCGTACAAGGCTCTAACCCACGCTTTCTTCAT 350

351 CTTCTAGGCTTCCGGTGGCAAGTATGTTCCCCGTGCCGTTCTGGTCGATC 400

401 TTGAGCCCGGTACCATGGACGCTGTCCGTGCCGGTCCCTTCGGTCAGCTT 450

451 TTCCGCCCCGACAACTTCGTCTTCGGCCAGTCTGGTGCCGGTAACAACTG 500

501 GGCCAAGGGTCACTA 515

Calmodulin Sequence of strain PET-4 (757 bp):

1 GTGTTCCATTCTTCCTGATGTAATGATGGGGAAACGAAGCGATCTTGCTT 50

51 TGTTTTCTACGACTTCAGCCTTATGGGAATATTCCAAGCTCACATGAGAT 100

101 TTTGCCTCCTCACAGGACAAGGATGGCGATGGTTAGTGCTATTCCGGTTT 150

151 CCCTTCCAATTCATCGACTCACGCGACCGGCTATTTTCCGTCGATATGGA 200

201 ATTTTTTTCACCCTGTTGCTTCCGGCGACCGATTTGCGACCAGGACGCTA 250

251 ATTTGTGAATTCACGCTGCAGGCCAGATCACCACCAAGGAGTTGGGCACT 300

301 GTTATGCGCTCGCTGGGCCAGAACCCCTCCGAGTCTGAGTTGCAGGACAT 350

351 GATCAACGAGGTTGACGCCGACAACAATGGCACCATCGATTTCCCCGGTA 400

401 TGCGATGATGGACACACCGATATCTGGAAAGGGAGACAATCCTGAAACTC 450

451 AGCTGCTAACCTCACGCAGAGTTCCTGACGATGATGGCCCGAAAGATGAA 500

501 GGATACCGATTCTGAGGAGGAAATCCGGGAAGCTTTCAAGGTCTTCGATC 550

551 GCGATAACAACGGTTTCATCTCGGCCGCGGAGCTGCGCCACGTCATGACC 600

601 TCCATCGGCGAGAAGCTCACCGACGACGAGGTCGATGAGATGATCCGTGA 650

651 GGCGGACCAGGATGGCGACGGCCGGATTGACTGTACGTTGTGACCTTGGG 700

701 ATGCCCTTTCTGAACCCAAACTAATGAACTGCTCCAGACAACGAGTTCAT 750

751 CCAACTC 757

Additional File 1: Figure S4 The received sequences of strain PET-4

ITS Sequence of strain PET-5 (525vbp)：

1 ACCTCCCACCCGTGTTTAACGAACCGTGTTGCTTCGGCGGGCCCGCCTCA 50

51 CGGCCGCCGGGGGGCATCCGCCCCCGGGCCCGCGCCCGCCGAAGCCCCCT 100

101 GTGAACGCTGTCTGAAGTATGCAGTCTGAGACAATTATTCAATTAATTAA 150

151 AACTTTCAACAACGGATCTCTTGGTTCCGGCATCGATGAAGAACGCAGCG 200

201 AAATGCGATAACTAATGTGAATTGCAGAATTCAGTGAATCATCGAGTCTT 250

251 TGAACGCACATTGCGCCCTCTGGTATTCCGGAGGGCATGCCTGTCCGAGC 300

301 GTCATTGCTGCCCTCCAGCCCGGCTGGTGTGTTGGGCCCCCGCCCCCCTT 350

351CCCGGGGGGGCGGGCCCGAAAGGCAGCGGCGGCACCGCGTCCGGTCCTCG 400

401 AGCGTATGGGGCTTTGTCACCCGCTCTTGCAGGCCCGGCCGGCGCCAGCC 450

451 GACCCCCTCAATCTATTTTTTCAGGTTGACCTCGGATCAGGTAGGGATAC 500

501 CCGCTGAACTTAAGCATATCAATAG 525

β-tubulin Sequence of strain PET-5 (420bp):

1 GGCACTCGAAGCAATATACTAACCAATTTTACAGGCAAACCATTGCCGGT 50

51 GAGCACGGCCTTGATGGCGATGGACAGTGAGTTCTTTAGACAACCTTTTG 100

101 ATTTTCGAGAATGGCGGTCTGATATTTTTGGGCAGGTACAACGGTACTTC 150

151 CGACCTCCAGCTGGAGCGCATGAACGTCTACTTCACCGAAGTAAGGGATC 200

201 TCGACATCAATTCTACTGACGATTCTCATTCTGACTGGTCGTTTCTTTTC 250

251 TCTCCAATAGGCTTCCGGTGACAAGTATGTTCCCCGTGCCGTTCTGGTCG 300

301 ATCTGGAGCCCGGTACCATGGACGCTGTCCGTGCCGGTCCTTTCGGCAAG 350

351 CTCTTCCGCCCCGACAACTTCGTCTTCGGTCAGTCTGGTGCTGGTAACAA 400

401 CTGGGCCAAGGGTCACTAAC 420

Additional File 1: Figure S5 The received sequences of strain PET-5
