# Supplementary material for: Production of theophylline via aerobic fermentation of pu-erh tea using tea-derived fungi
Source: BMC Microbiol. 2019 Nov 26;19:261. doi: 10.1186/s12866-019-1640-2 (PMC6878699; doi:10.1186/s12866-019-1640-2)
Supplement: Supplementary file 2 — Additional file 2 Table S1. Changes of other main chemical compounds in the fermentation of A. sydowii PET-2. [file 12866_2019_1640_MOESM2_ESM.doc]

Additional file 2:Table S1

Changes of other main chemical compounds in the fermentation of *A. sydowii* PET-2

| Time (d) | Tea polyphenols (mg/g) | Theaflavins (mg/g) | Thearubigins (mg/g) | Theabrownins (mg/g) |
| --- | --- | --- | --- | --- |
| 0 | 355±7^e^ | 2.47±0.11^a^ | 22.68±1.11^a^ | 18.6±1.1^a^ |
| 3 | 325±9^d^ | 3.13±0.26^b^ | 31.33±2.65^b^ | 40.5±5.0^b^ |
| 6 | 288±8^c^ | 3.46±0.08^c^ | 33.80±2.19^b^ | 58.5±6.3^b^ |
| 9 | 279±6^c^ | 4.02±0.12d | 38.87±2.95^c^ | 78.6±11.8^c^ |
| 12 | 255±8^b^ | 4.47±0.11^e^ | 41.40±2.87^c^ | 101.5±16.6^d^ |
| 15 | 221±4^a^ | 4.54±0.30^e^ | 42.79±2.65^c^ | 109.7±13.4^d^ |

Tea polyphenols content was determined by using the spectraphotometric method based on FeSO_4_. Contents of the main tea pigments, including theaflavins, thearubigins and theabrownins were analyzed by using the spectrophotometric method. All data are presented as mean value ± SD of three replications. The lowercase letters show significant differences at *p* < 0.05 level (Duncan`s multiple range test), and the different letters show significant differences. Samples collected on 0 day were the raw material for the fermentation.
